# The Human Cathelicidin Antimicrobial Peptide LL-37 as a Potential Treatment for Polymicrobial Infected Wounds

**DOI:** 10.3389/fimmu.2013.00143

**Published:** 2013-07-03

**Authors:** Allen J. Duplantier, Monique L. van Hoek

**Affiliations:** ^1^National Center for Biodefense and Infectious Diseases, George Mason University, Manassas, VA, USA; ^2^School of Systems Biology, George Mason University, Manassas, VA, USA

**Keywords:** antimicrobial peptide, chronic wounds, biofilm, infected wounds, cathelicidin

## Abstract

Diabetic patients often have ulcers on their lower-limbs that are infected by multiple biofilm-forming genera of bacteria, and the elimination of the biofilm has proven highly successful in resolving such wounds in patients. To that end, antimicrobial peptides have shown potential as a new anti-biofilm approach. The single human cathelicidin peptide LL-37 has been shown to have antimicrobial and anti-biofilm activity against multiple Gram-positive and Gram-negative human pathogens, and have wound-healing effects on the host. The combination of the anti-biofilm effect and wound-healing properties of LL-37 may make it highly effective in resolving polymicrobially infected wounds when topically applied. Such a peptide or its derivatives could be a platform from which to develop new therapeutic strategies to treat biofilm-mediated infections of wounds. This review summarizes known mechanisms that regulate the endogenous levels of LL-37 and discusses the anti-biofilm, antibacterial, and immunological effects of deficient vs. excessive concentrations of LL-37 within the wound environment. Here, we review recent advances in understanding the therapeutic potential of this peptide and other clinically advanced peptides as a potential topical treatment for polymicrobial infected wounds.

## Introduction

The goal of this review article is to explore and analyze in-depth the recent published literature and ongoing clinical trials that have focused on the potential of the antimicrobial peptide (AMP) LL-37 to be used in infected wound treatment, especially as a potential topical treatment. We are especially concerned with the treatment of wounds that may contain multiple, biofilm-forming organisms (polymicrobial infections). Many significant technical and clinical advances have been made in this area, and we saw a need for an overview of the current state of the art. In addition, we sought to link the effects of LL-37 on the pathogen with its concomitant effects on the host in the infected wound model system, as this potentially may be a synergistic activity that illustrates the benefits of LL-37 (or derivatives) as a potential therapeutic agent for the topical treatment of infected wounds. Finally, we sought to identify some of the remaining challenges that exist in bringing such a treatment to patients.

**Figure 1:**
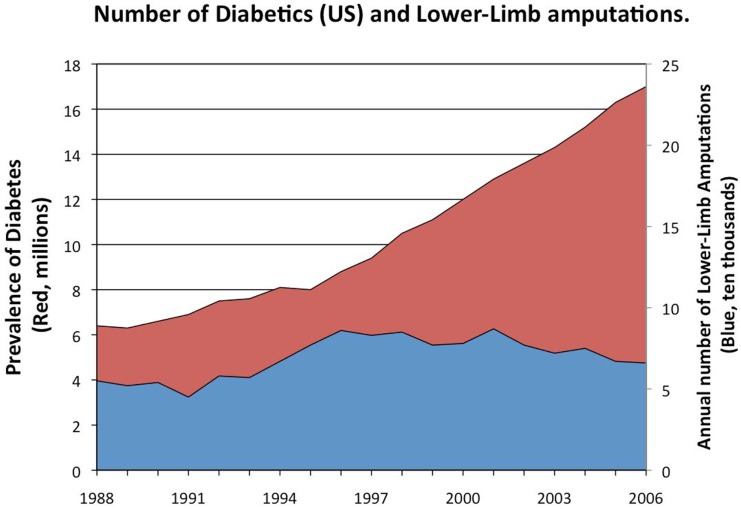
**Prevalence of diabetes in the US (red) and the number of lower-limb amputations**. Between 15 and 25% of diabetics will develop diabetic foot ulcers in their lifetime. Diabetics often suffer from severe, chronic infections of those ulcers (James et al., 2008; Wolcott et al., 2008, 2010a; Fisher et al., 2010) leading to as many as 70,000 lower-limb amputations per year in the United States (Wolcott et al., 2010a). These biofilm-associated infections and their sequelae greatly contribute to the healthcare cost for diabetic patients; as much as $174 billion is spent annually on diabetes in the United States (Mikkelsen et al., 2011). With almost 26 million diabetics (8.3% of the population) in the US currently and the number expected to rise, the number of these life-threatening infections is likely to increase.

Bacterial biofilms inhibit wound healing and promote infection. Opportunistic pathogens, such as *Pseudomonas aeruginosa* and *Staphylococcus aureus* are able to infect open wounds such as chronic diabetic foot ulcers (Johnson et al., 2007; James et al., 2008; Murray, 2008a,b; Ressner et al., 2008; Wolcott et al., 2008, 2010a; Brown et al., 2010; Fisher et al., 2010). These organisms have a prodigious ability to produce biofilm that makes eliminating them from wounds extremely challenging. The immune system can be ineffective against the infections as a result of poor circulation particularly in diabetic patients. Antibiotics can be ineffective due to lack of penetration through the biofilm or due to colonization by resistant strains and poor circulatory delivery of systemic antibiotics. The result can be chronically infected wounds with polymicrobial bacterial populations that threaten the lives and limbs of patients (Lopez-Leban et al., 2010). The current approach to controlling these severe infections in diabetic patients includes performing 70,000 lower-limb amputations every year in the USA (Figure 1) (Wolcott et al., 2010a). With 26 million (and rising) diabetics in the US, these life-threatening infections are likely to increase. There are few new antibiotics in the drug-development pipeline that are effective against *Pseudomonas* and *Staphylococcus* within these wounds. A combination approach of wound-care management (debridement), systemic antibiotics, plus the use of topical anti-biofilm agents (e.g., xylitol) has been shown to reduce the ability of the biofilm to persist (Wolcott and Rhoads, 2008; Lopez-Leban et al., 2010; Wolcott et al., 2010a,b,c) and has been shown to be one effective approach to healing these wounds. Once the biofilm collapses, the infecting bacteria are unprotected and then cleared by the immune system and antibiotics. AMPs are a potential new topical therapeutic agent to include in this combinatorial approach particularly due to their anti-biofilm activity at low concentrations. Humans make a single cathelicidin AMP, LL-37, which has both antimicrobial and anti-biofilm properties and can eradicate preformed biofilms *in vitro* (Overhage et al., 2008; Dean et al., 2011a,b). Thus, there is great interest of LL-37 as a potential therapeutic for polymicrobial infected wounds. In this review, we will survey recent research on the host and pathogen targets of this peptide, and its potential for use in the treatment of polymicrobial infected wounds.

### Biofilms in wounds

**Figure 2:**
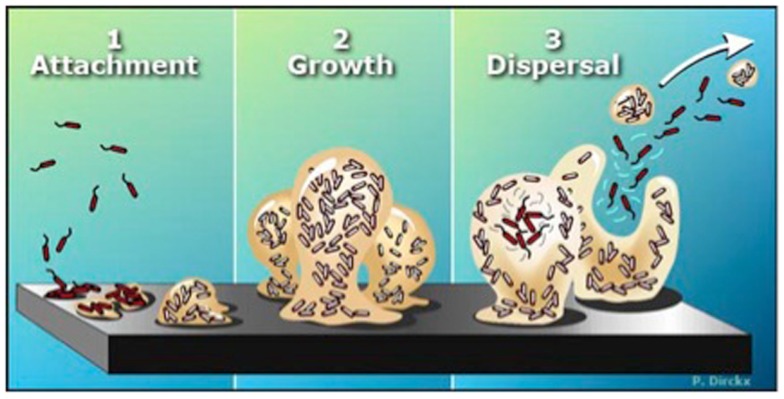
**The biofilm life cycle**. (1) Free-floating (planktonic) bacteria encounter a submerged surface and within minutes can become attached. They begin to produce slimy extracellular polymeric substances (EPS) and start to colonize the surface. (2) EPS production allows the emerging biofilm community to develop a complex, three-dimensional structure that is influenced by a variety of environmental factors. Biofilm communities can develop within hours. (3) Biofilms can propagate through detachment of small or large clumps of cells, or by a type of “seeding dispersal” that releases individual cells. Either type of detachment allows bacteria to attach to a surface or to a biofilm downstream of the original community. [Biofilms: The Hypertextbook (http://www.hypertextbookshop.com/biofilmbook/v004/r003/index.html). Used with permission from Montana State University Center for Biofilm Engineering].

Bacteria prevailingly exist within biofilms – sessile communities of microorganisms that synthesize and surround themselves with a slimy, hydrated polymeric matrix attached to a solid surface. Biofilm-forming bacteria (while in the planktonic state) go through an initial “twitching” stage where they explore a surface prior to attachment in a Type IV-pili dependent manner. Once irreversibly attached, the bacteria begin to grow, differentiate and excrete a hydrated polymeric matrix within which a micro-colony of bacteria is formed (Figure 2). The micro-colony then uses quorum-sensing molecules to communicate from cell to cell. While embedded in this matrix, bacteria exhibit an altered phenotype with respect to growth rate and gene transcription (Costerton et al., 1995; Wolcott et al., 2010b). The transition of planktonic bacteria from a free-swimming mode (using flagella in the case of *Pseudomonas*) to the formation of (and growth within) a biofilm environment attached to a surface is described in Figure 2 and has been well reviewed (Monds and O’Toole, 2009; Abee et al., 2011; Mikkelsen et al., 2011; Petrova and Sauer, 2012). Once within a biofilm, bacteria can thrive as they are protected from the immune system, nutrient deprivation, changes in pH, and antimicrobial agents (Monds and O’Toole, 2009). Growth within the biofilm eventually reaches its maximum and is followed by dispersion of the bacteria from the mature biofilm, believed to be induced, in part, by short-chain fatty acid signaling molecules such as *cis*-2-decenoic acid (Davies and Marques, 2009).

Chronic infected wounds may result from pressure sores, venous leg ulcers, diabetic foot ulcers, burns, surgical site infections, combat wounds, and other factors, suffer from persistent inflammation and are often infected with strong biofilm-forming bacteria (James et al., 2008). Adding to the complexity is the finding that these infections are often composed of multiple microbial species attacking host cells, and thus antibiotic therapy that target specific bacteria becomes ineffective (Dowd et al., 2008). Moreover, the emergence of multi-drug-resistant strains of wound containing bacteria such as *S. aureus* (e.g., methicillin-resistant *S. aureus*, MRSA, and *Acinetobacter baumannii*) has intensified the need for new treatments.

**Figure 3:**
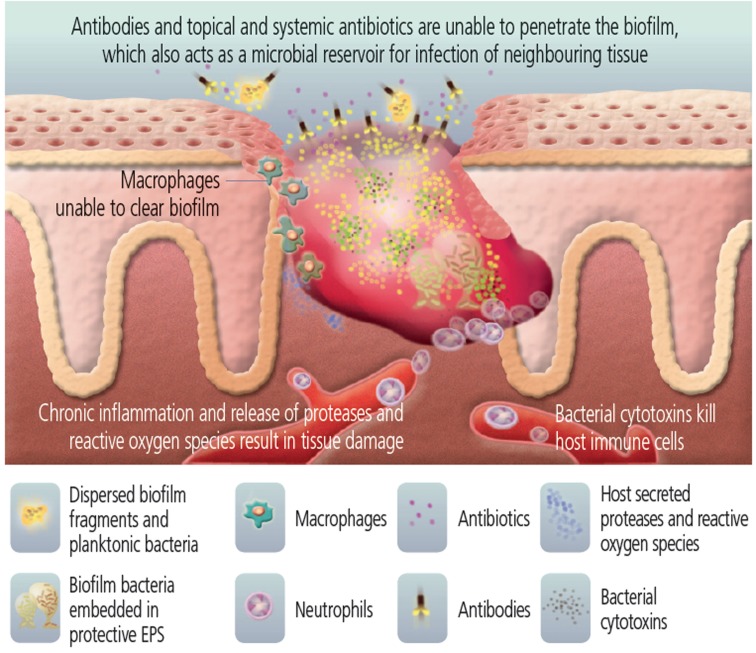
**Bacteria in the wound are protected by biofilm**. [Image used with permission from Biofilms Made Easy, 2010, Vol. 1, Issue 1, published on Wounds International (http://www.woundsinternational.com/pdf/content_8851.pdf)].

Growing evidence supports the hypothesis that the presence of biofilm actively prevents the healing of these wounds (Figure 3) (Wolcott et al., 2010b). While physical debridement can assist the healing of chronic infected wounds, anti-biofilm approaches in combination with anti-inflammatory and antimicrobial therapy may promote more rapid healing in a broad range of chronic wound patients (Wolcott et al., 2009; Ammons, 2010). Wolcott and Dowd have shown in mouse models and human patients that elimination of biofilm from a wound promotes wound healing (James et al., 2008; Wolcott et al., 2008, 2010c; Dowd et al., 2009; Fisher et al., 2010; Lopez-Leban et al., 2010). The use of multiple concurrent strategies to treat these wounds is most effective, combining physical debridement, systemic antibiotics, and topical treatments that reduce biofilm. Once the biofilm collapses, the host immune system and the systemic antibiotics are able to combat the unprotected bacteria, the infection resolves, and the wound heals (Wolcott and Rhoads, 2008; Lopez-Leban et al., 2010; Wolcott et al., 2010a,b,c), even though diabetic patients often have impaired wound healing as part of the disease process.

Thus, the use of an anti-biofilm agent may represent an effective strategy to treat chronic infected wounds by enabling innate and adaptive immunity, as well as concomitant treatment with existing antibiotics to become more effective.

### Antimicrobial peptides

Antimicrobial peptides are essential components of human innate immunity and contribute to the first line of defense against infection (Zasloff, 2002). There are currently over 1480 known AMPs with antibacterial, anticancer, antiviral, and antifungal activities (Wang et al., 2009). In general, many of these AMPs are cationic, amphipathic, between 12 and 100 amino acids in size, are structurally diverse and are capable of binding to and disrupting the membranes of microbes (Yeaman and Yount, 2003; Hurdle et al., 2011). AMPs can inhibit cell wall, nucleic acid, and protein biosynthesis (Brogden, 2005) and are chemotactic for many leukocytes, drawing them to the site of infection or inflammation. They have also been shown to be capable of binding and neutralizing lipopolysaccharides (LPS), promoting angiogenesis and wound healing, and exerting anti-tumor activity. Even though AMPs have co-evolved with bacteria over millions of years, bacteria have surprisingly not developed wide-spread resistance, providing AMPs with a potentially attractive advantage over existing antimicrobial agents.

### Cathelicidins

The cathelicidin family of AMPs, named by the ability to inhibit the protease cathepsin-L, is a large and diverse group of peptides containing a conserved N-terminal domain called the cathelin domain. Cathelicidins can be found in their precursor form in the granules of natural killer T cells, neutrophils, and in the mucosal epithelia of the lungs, with the functional antimicrobial cathelicidin peptide generated through proteolytic removal of the cathelin domain as part of the secretion process (Bals, 2000). This class of peptides has been shown by us and many other researchers to be antimicrobial against many human bacterial pathogens (Amer et al., 2010; de Latour et al., 2010; Dean et al., 2011a,b; Jiang et al., 2011; Kanthawong et al., 2012). The sequence diversity of cathelicidins resides in the active peptide following cleavage of the conserved N-terminus, and thus cathelicidins are structurally conserved AMPs containing amphipathic α-helices without sequence conservation.

In humans, the cathelicidin gene encodes an inactive 18 kDa precursor protein (Hcap-18) that releases the active C-terminus 37 amino acid peptide LL-37 (LLGDFFRKSKEKIGKEFKRIVQRIKDFLRNLVPRTES) upon processing. Overviews of the structural properties, expression, cellular and tissue distribution, and antimicrobial, chemotactic, and immunomodulatory activities of this intriguing class of peptide have been published (Durr et al., 2006; Kai-Larsen and Agerberth, 2008; Burton and Steel, 2009; Nijnik and Hancock, 2009; Mendez-Samperio, 2010; Jacobsen and Jenssen, 2012; Vandamme et al., 2012). This review will focus on the recent reports of LL-37 and its interactions with bacteria, biofilm, and host cells making it a potentially effective agent for the treatment of chronic non-healing wounds.

## LL-37 Peptide

### LL-37 structure

In order to begin to understand how LL-37 interacts with biofilms, bacteria, and host cells, it is important to consider its features and secondary structure. Fifty-four percent of LL-37’s residues are hydrophilic with 11 basic and 5 acidic, giving it a net positive charge of +6 at physiological pH. In aqueous solution LL-37 exhibits a circular dichroism spectrum that is consistent with a disordered structure (see Table 1, entry A). However, in membranes where the environment is lipophilic, many of the amino acids are able to form intramolecular hydrogen bonds (backbone N–H groups donate a hydrogen bond to the backbone C=O groups that are four amino acids earlier in the sequence) locking the secondary structure into an α-helix (Dean et al., 2011a).

**Table 1:** **Secondary structure of LL-37**.

	LLGDFFRKSKEKIGKEFKRIVQRIKDFLRNLVPRTES	LL-37 sequence
**A**	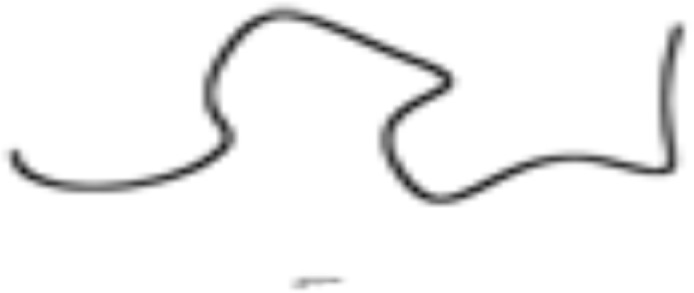	LL-37 in aqueous media has no defined conformation

**B**	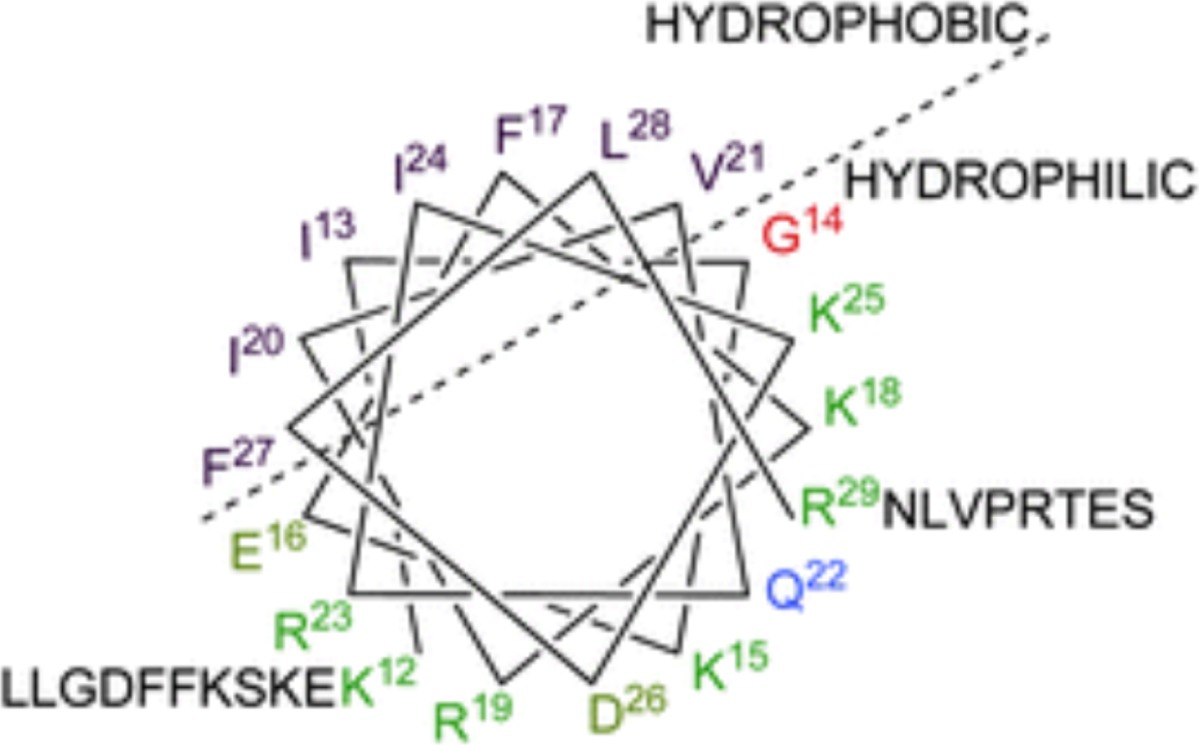	Helical wheel diagram for LL-37 showing the region 12–29 as an amphipathic helix. N- (residues 1–11) and C- (residues 30–37) termini residues are unstructured (Burton and Steel, 2009)

**C**	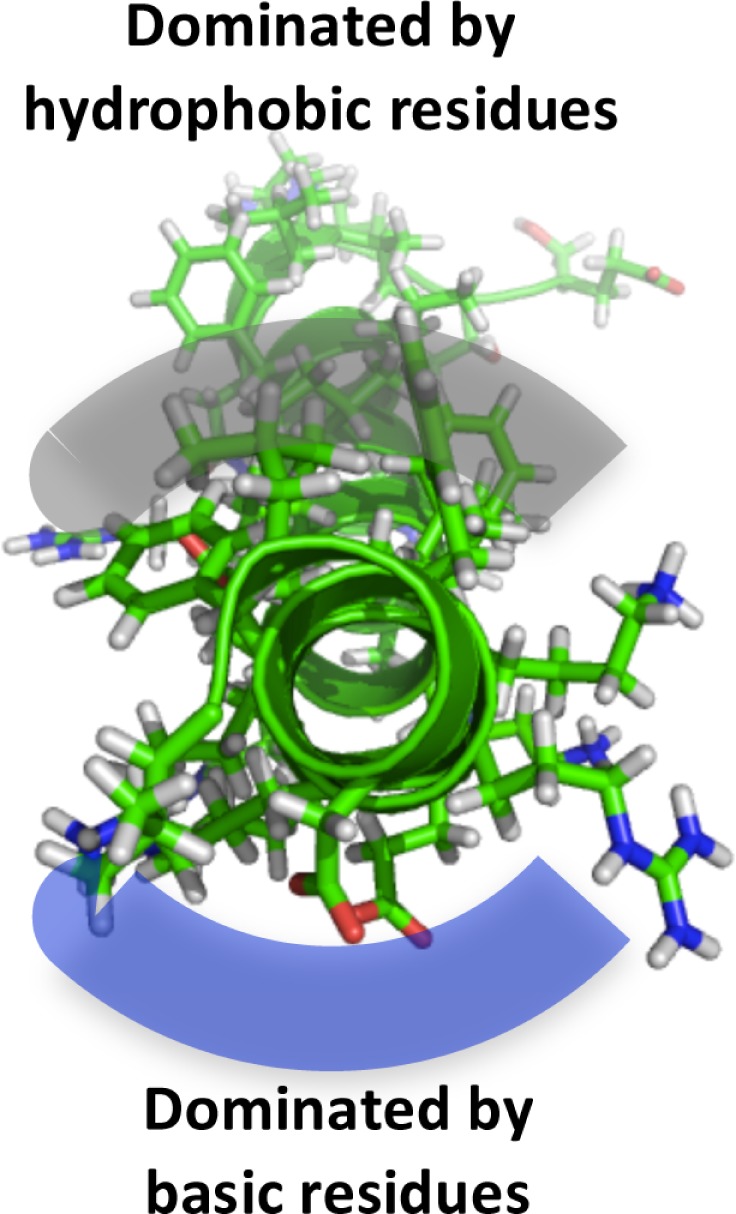	View of AMP looking through the coil showing its amphipathic nature LL-37 structure from PDB 2K6O. Image generated using MacPyMol

**D**	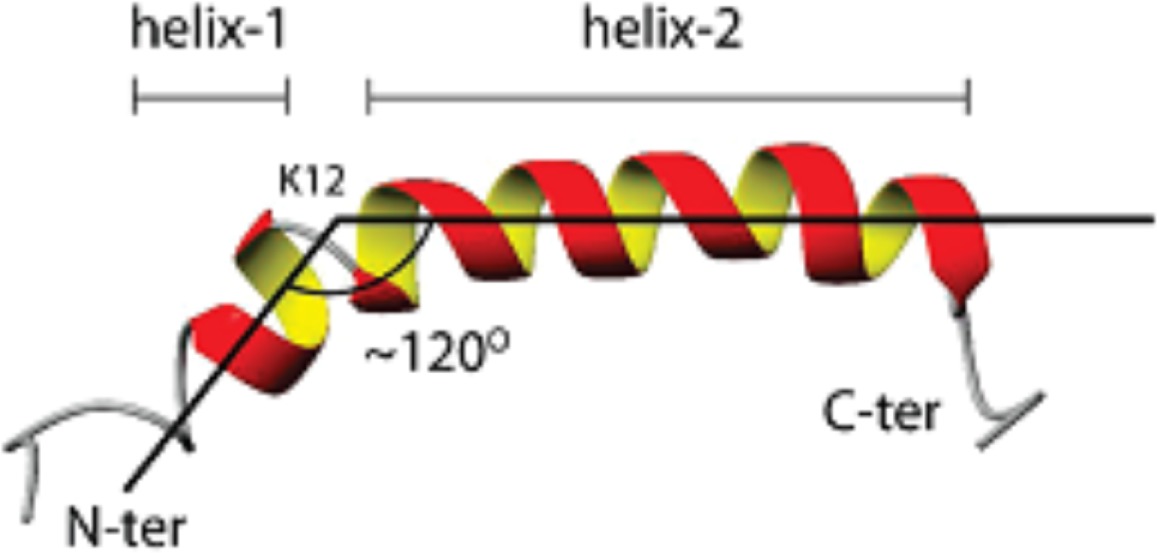	NMR structure of LL-37 in dodecylphosphocholine micelles showing the angle between the two helical domains and the break point centered at K12 (Porcelli et al., 2008)

**E**	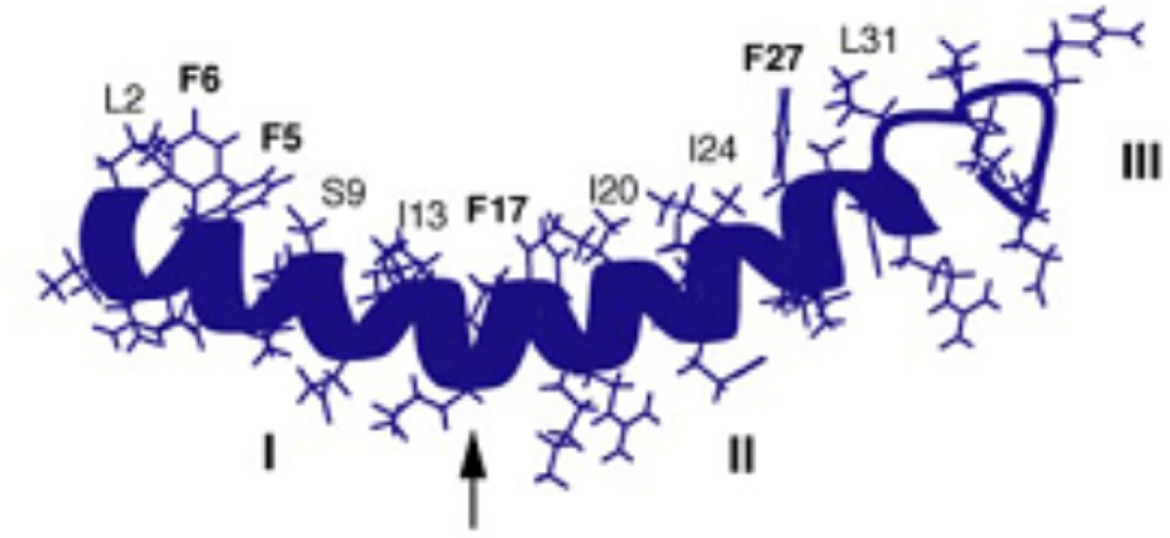	NMR structure of LL-37 in SDS micelles with hydrophobic side chains labeled. The helical bend is indicated by an *arrow*, and the three structural regions are labeled with Roman numerals I, II, and III (Wang, 2008)

A characteristic feature of the LL-37 α-helix is its amphipathic nature, illustrated by the helical wheel and molecular model diagrams in Table 1, entries B and C, respectively. The secondary structure, while viewing into the coil, reveals a lipophilic side and a polar side that is positively charged (cationic) at physiological pH (7.4). In efforts to more accurately approximate the conformation of LL-37 in membranes, the three-dimensional NMR structure was elucidated in dodecylphosphocholine micelles (Porcelli et al., 2008). Under these conditions, both the N- and C-termini were unstructured and solvent exposed, and the N- and C-terminal helixes were hinged at K12, supported by a hydrophobic cluster formed by I13, F17, and I20, and a salt bridge between K12 and E16 (see Table 1, entry D). The hydrophilic face of LL-37 was exposed to the water phase and the hydrophobic face was buried in the micelle hydrocarbon region. In a similar fashion, a three-dimensional triple-resonance NMR spectroscopy study of LL-37 in SDS micelles was performed (Wang, 2008). Under these conditions a curved amphipathic helix-bend-helix motif was found that spanned residues 2-31, and the C-terminal tail was disordered (Table 1, entry E). The extent of α-helicity appears to correlate with the antibacterial activity of LL-37 against both Gram-positive and Gram-negative bacteria (Johansson et al., 1998).

Structural information along with structure activity relationships around LL-37 and its smaller fragment derivatives continue to emerge. For a comprehensive review of the sequence and bioactivity of published native fragments and synthetic analogs of LL-37 (see Burton and Steel, 2009). A number of these smaller peptidic analogs have similar antimicrobial activities compared with LL-37, but are less cytotoxic and more stable in the presence of serum (Ciornei et al., 2005), and thus may be useful tools for evaluating AMPs in the treatment of chronic infected wounds.

### LL-37 endogenous levels

The LL-37 peptide is produced by proteolytic cleavage (Protease 3) of hCAP-18 that exists at high concentrations in the granules of neutrophils (40 μM or 630 μg 10^9^ cells) (Sørensen et al., 1997, 2001). However, in skin the serine proteases SCTE (stratum corneum tryptic enzyme, kallikrein 5), SCCE (stratum corneum chymotryptic enzyme, kallikrein 7), and kallikrein-related peptidase-8 (KLK8) were shown to control activation of hCAP-18 and influence further processing to smaller peptides with alternate biological activity (Yamasaki et al., 2006; Eissa et al., 2011). In support of this finding, doxycycline and other matrix metalloproteinase inhibitors were recently found to inhibit the generation of LL-37 from hCAP-18 in keratinocytes, a process dependent on kallikrein activity (Kanada et al., 2012). The physiological concentration of LL-37 varies within different tissues and cells, and is often altered at sites of infection. For example, LL-37 levels were significantly elevated in serum specimens from multiply injured patients (0.02 ∼0.04 vs. 0.002 μM for controls) (Lippross et al., 2012), and LL-37 was highly expressed in the skin of psoriasis patients (Reinholz et al., 2012). More relevant to chronic infected wounds, LL-37 expression was lower in keloid and atopic dermatitis patients compared with normal (Park et al., 2009), and in the epidermis of diabetic foot ulcers and chronic venous ulcers LL-37 had little to no expression compared to healthy skin (Rivas-Santiago et al., 2012). This later data may imply that part of the issue contributing to the chronic infection present in non-healing wounds may be the low levels of the innate immune system peptide LL-37.

Unbound LL-37 levels in the wound are regulated by a balance of expression, degradation, and serum (or wound exudate) protein binding. Adding to the complexity, the pathogen itself, through LPS found in the outer membrane of Gram-negative bacteria can induce the expression of LL-37. This induction occurs via interaction at the toll-like receptor 4 (TLR4) and through subsequent release of cytokines such as IL-4, IL-5, IL-1β, and TNF-α. As a counteraction, LL-37 in turn can bind to LPS and prevent its interaction with lipopolysaccharide-binding protein (LBP) and the co-receptor CD14, thus neutralizing the effect of LPS (Nagaoka et al., 2001; Yoshioka et al., 2008). In general, the release of the LL-37 peptide in the wound is a physiological response to host/pathogen induced TLR and NOD-like receptor (NLR) signals (Thoma-Uszynski et al., 2001).

LL-37 antibacterial activity was found to be unaffected by the presence of 50% serum, citrate-plasma, or EDTA-plasma. However, heparin-plasma and wound fluids that contain glycosaminoglycans (GAGs) (e.g., dermatan sulfate) attenuated the antibacterial effects of LL-37. These effects could be abrogated by the addition of polycationic polysaccharides (e.g., chitosan) that form complexes with GAGs (Baranska-Rybak et al., 2006). In infected wounds, bacteria such as *P. aeruginosa* were able to release GAGs from connective tissues and block the bactericidal actions of LL-37. In addition, *P. aeruginosa* elastase is a secreted protease that has been shown to further deactivate LL-37 by cleaving it at many of the amino acid junctions (Schmidtchen et al., 2002). Within host cells, mast cell activation by LL-37 results in the release of mast cell protease (β-tryptase) that degrades and inactivates LL-37. However, in this case as a counter-regulation, the platelet-derived chemokine, CXCL4, protects LL-37 from cleavage by β-tryptase by antagonizing the heparin component required for the integrity of the active tetramer of β-tryptase (Schiemann et al., 2009).

**Figure 4:**
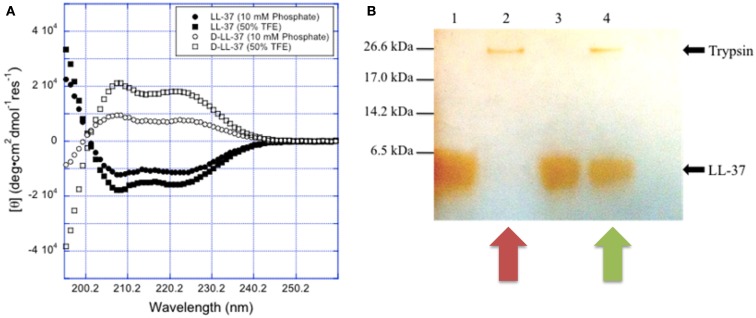
**Chirality affects LL-37 susceptibility to proteases**. **(A)** The spectra for l- and d-LL-37 (125 μM) exhibit significant helical character in 10 mM sodium phosphate buffer (pH = 7.4). As expected, the spectrum for d-LL-37 (∙) is the mirror image of that of the l-peptide (∘). The spectra for both d- and l-LL-37 (□ and n respectively) become more intense when the peptides are in 50% TFE in 10 mM phosphate buffer (pH = 7.4), consistent with the peptides exhibiting more helical character in the presence of these membrane-mimics. These results are consistent with what has been reported in the literature for these peptides. **(B)** d-LL-37 demonstrated resistance to degradation by trypsin. Peptides (18 μg) were dissolved in water (90 μL) with either water or 0.05% trypsin (10 μL) and incubated (37 °C, 1 h). Ten microliters of aliquots were separated on SDS-PAGE, and a silver stain was performed. Lane 1, LL-37; Lane 2, LL-37 with trypsin; Lane 3, d-LL-37; Lane 4, d-LL-37 with trypsin. (Figure from Dean et al., 2011a, used with permission).

Noteworthy, since protease cleavage typically occurs between natural amino acids, the incorporation of unnatural amino acids can be used as a strategy to improve metabolic stability. For example, the unnatural enantiomer d-LL-37 (in which each amino acid is in the d-configuration) was found to be resistant to trypsin degradation (Figure 4) (Dean et al., 2011a). In *ex vivo* experiments, LL-37 was degraded by trypsin, but its susceptibility to trypsin was diminished in the presence of wound fluid up to 24 h (Gronberg et al., 2011). Thus, the protein binding of LL-37 in wound fluid may protect it from protease degradation. Regardless, there may be significant benefit to using protease-resistant analogs of LL-37 *in vivo* to prolong the effective half-life. To that end, we have performed *in vivo* studies using the wax moth caterpillar and demonstrated an increased survival of *Pseudomonas* infected caterpillars following d-LL-37 treatment (Figure 6) (Dean et al., 2011a). These results suggest that d-LL-37 may be more effective at rescuing caterpillars from *Pseudomonas* infection, likely due to its improved protease resistance.

In leukocytes and keratinocytes, the mechanisms regulating LL-37 production have been linked to vitamin D3. Induction of LL-37 by 1,25-dihydroxyvitamin D3 requires the intracellular vitamin D receptor (VDR), as well as the steroid receptor coactivator 3 (SRC3) and histone acetylation (Schauber et al., 2008). Thus, the host/pathogen response to the regulation of LL-37 is complex.

### LL-37 mechanism of interaction with microbial membranes

**Figure 5:**
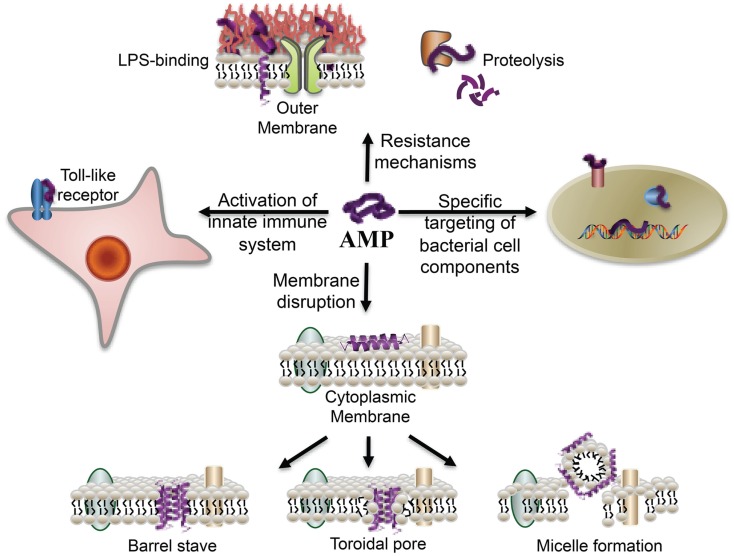
**Overview of the broad-spectrum of cellular interactions associated with antimicrobial peptides**. In addition to exerting antimicrobial activity by disrupting bacterial membranes, peptides may also bind to specific target proteins within microbial cells and activate the innate immune system. The binding of peptides to cell-surface LPS molecules and proteolysis contribute to bacterial resistance to AMPs (From Marsh et al., 2009, reproduced by permission of the Royal Society of Chemistry).

LL-37 has broad-spectrum antimicrobial activity against both Gram-negative and Gram-positive bacteria (Durr et al., 2006) including drug-resistant strains (Saiman et al., 2001; Zaiou et al., 2003; Schittek et al., 2008). AMPs, and LL-37 in particular, have a different mode of action compared to conventional antibiotics as its size, shape, lipophilicity, and cationic nature interacts with the lipophilic and anionic nature of LPS, a component of the outer membrane of most Gram-negative bacteria (Figure 5). Using fluorescence microscopy, the antimicrobial activity of LL-37 attacking the Gram-negative bacteria *E. coli* was recently dissected into stages (Sochacki et al., 2011). The first stage was binding to the outer membrane and its LPS and O-antigen layers, which quickly saturate. At 8 μM, LL-37 binding saturated the outer membrane within 1 min. Translocation across the outer membrane and access to the periplasmic space correlated in time (5–25 min later) with the halting of growth, which may occur because of LL-37 interference with cell wall biogenesis. As shown in Figure 5, after membrane association there are several proposed models of how the AMP may induce bacterial killing (for an overview see Brogden, 2005). In the barrel-stave model, the LL-37 peptides would attach, aggregate, and insert into the inner membrane bilayer with the hydrophobic side of the peptide aligning with the lipid core region and the hydrophilic side forming the interior region of the pore (Table 1, B and C). In the Toroidal model, the attached LL-37 peptides would aggregate and bend the lipid monolayers continuously through the pore so that the core would become lined by the head groups of both the inserted peptides and the lipid monolayer. In the carpet model (micelle formation), the LL-37 peptides would disrupt the membrane by orienting parallel to the surface of the lipid bilayer and form an extensive layer or carpet. In all cases, the interaction would result in pores being formed within the inner membrane followed by bacterial lysis.

**Figure 6:**
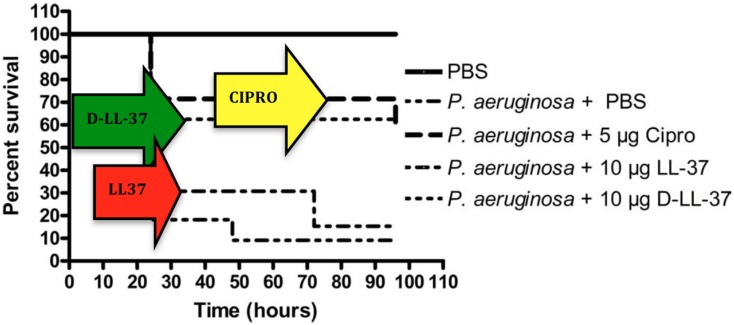
**Treatment of *P. aeruginosa*-infected *G. mellonella***. d-LL-37 significantly prolonged the survival of *G. mellonella* infected with 1 × 10^3^ CFU *P. aeruginosa*. Non-infected control groups consisted of a PBS injection, 5 μg Ciprofloxacin injection, 10 μg LL-37 injection, or a 10 μg d-LL-37 injection. All non-infected groups fare similarly to the PBS only group (data not shown). The non-infected PBS only group experienced the highest survival rate and was significantly different from all other groups (*p*-value < 0.002). The infected group without treatment failed to survive beyond 24 h. A single dose of 5 μg ciprofloxacin, or 10 μg of either d- or l-LL-37 was found effective when compared to the infected control group (*p*-value < 0.01), but not different from each other overall. At 48 h d-LL-37 was found to be more effective than l-LL-37 (*p*-value < 0.04). (Figure from Dean et al., 2011a, used with permission).

LL-37 and its LL-31 truncated analog (lacking the 6 C-terminus residues) exhibited a strong killing effect against *Burkholderia pseudomallei* (Kanthawong et al., 2012). The percentage of α-helical structure as determined by circular dichroism was similar for LL-37 vs. LL-31. In this study, the killing of *B. pseudomallei* (as well as *B. thailandensis)* was shown to be caused by disruption of membrane as measured by freeze-fracture electron microscopy of bacterial cells. Both peptides exhibited stronger antimicrobial activity against *B. pseudomallei* in biofilm compared to ceftazidime, a cephalosporin antibiotic that is used clinically for initial melioidosis treatment. This result is consistent with LL-37’s ability to permeabilize and/or to form pores within the cytoplasmic membrane.

In recent studies, LL-37 has also been shown to have specific binding interactions with the outer membrane lipoprotein Lpp in *Enterobacteriaceae* (Chang et al., 2012). Lpp is composed of trimeric α-helices (in aqueous solution) (Shu et al., 2000), and although proposed to act as a barrier against antibiotics, there is evidence that LL-37 binds and internalizes Lpp. The crystal structure of Lpp provides an explanation for assembly and insertion of the lipoprotein molecules into the outer membrane of Gram-negative bacteria. The authors suggest that the susceptibility of bacteria to an AMP is not strictly correlated with the presence of Lpp on bacteria, as the bactericidal activities were blocked by anti-Lpp antibodies. As specific receptors and mechanisms for which LL-37 interacts with bacteria are gradually becoming understood, it is clear that further research is warranted in the area.

Finally, for some AMPs, there is a mechanism by which the peptide can penetrate the bacterial cell and potentially act directly on intracellular targets, though primarily to be nucleic acids (DNA or RNA) (Takeshima et al., 2003; Lan et al., 2010; Madani et al., 2011). Recent studies suggest that LL-37 may be able to be a cell-penetrating peptide as well (Zhang et al., 2010; Hoyer et al., 2012).

### LL-37 anti-biofilm activity

One of our long term goals is to discover new treatments for polymicrobial infected wounds, which are often biofilm-mediated (Edwards and Harding, 2004). These wounds are most often infected with multiple bacteria (polymicrobial) and usually produce a large amount of biofilm as part of the pathology of the infection. New treatments for non-healing infected chronic wounds are a high need, especially in light of emerging antibiotic resistant organisms.

The mechanism by which LL-37 carries out its anti-biofilm effect is unknown for the case of *S. aureus*, and is suggested as the dysregulation of biofilm regulatory systems and quorum-sensing in *P. aeruginosa* (Overhage et al., 2008). Thus, several mechanisms for anti-biofilm activity are possible. For example: prevention of twitching and/or the initial attachment; membrane perturbation leading to an SOS response (Coenye, 2010); and blocking intracellular quorum-sensing molecules. Interestingly, LL-37 potently inhibited the formation of bacterial biofilms, including *S. aureus* (Dean et al., 2011b), and *P. aeruginosa in vitro* at concentrations (0.5 μg/ml) far below that required to kill or inhibit bacterial growth (MIC 64 μg/ml) (Overhage et al., 2008; Dean et al., 2011a). In this example the anti-biofilm activity was demonstrated to be mediated in three ways: (a) reduction of the initial attachment of *P. aeruginosa* cells to the surface; (b) promotion of twitching by stimulating the expression of genes related to type IV pilus biosynthesis and function (increased surface motility would cause bacteria to wander across the surface instead of forming biofilms); and (c) down-regulation of key components of the Las and Rhl systems (quorum-sensing systems of *P. aeruginosa*). The ability of LL-37 to inhibit biofilm formation, especially at physiologically relevant concentrations, is a promising feature with regards to the treatment of chronically infected wounds.

The connection between biofilm formation and hard-to-heal wounds was demonstrated in a murine cutaneous wound model where *Staphylococcal* biofilms were shown to delay re-epithelialization. In this experiment, the disruption of the quorum-sensing system through the addition of RNAIII inhibiting peptide (RIP) (breaking the cycle of biofilm signaling) restored normal wound healing (Schierle et al., 2009). It is important to point out that even though RIP is neither bactericidal nor bacteriostatic (Kiran et al., 2008), it reduced the ability of bacteria to survive within the host. In other words, “when the biofilm collapses, the once protected bacterial community can be targeted by the immune system and by antibiotics, allowing complete recovery of the otherwise non-healing wound” (Wolcott et al., 2011). With respect to the LL-37 peptide, in addition to its ability to be anti-biofilm and antimicrobial, it plays an important role in regulating the balance of pro- and anti-inflammatory molecules both under homeostatic conditions and during bacterial or endotoxin challenge (Mookherjee et al., 2006). LL-37 peptide thus demonstrates broad-spectrum antimicrobial and anti-biofilm properties (Overhage et al., 2008; Dean et al., 2011a,b), making it a strong candidate to develop into a topical therapeutic for infected combat or burn wounds or chronic, non-healing wound such as diabetic ulcers.

Methods for screening new molecules for anti-biofilm activity in an *in vitro* model of an infected wound are available. For example, the Lubbock chronic wound biofilm model is an *in vitro* multispecies biofilm model that simulates the functional characteristics of chronic pathogenic biofilms (Sun et al., 2008). Visually, as well as by electron microscopy, this model is morphologically similar to actual chronic wound biofilms and shows promise as a tool for discovering new anti-biofilm agents.

It may be more appropriate to refer to these peptides as “anti-biofilm peptides” rather than “AMPs,” reflecting our current understanding of the potential role of biofilms in infection. Incorporation of anti-biofilm peptides or their synthetic derivatives in therapeutic topical applications may improve outcomes for infections ranging from chronic wounds, burns, implanted medical devices, and pneumonia.

### LL-37 interaction with host cells

**Figure 7:**
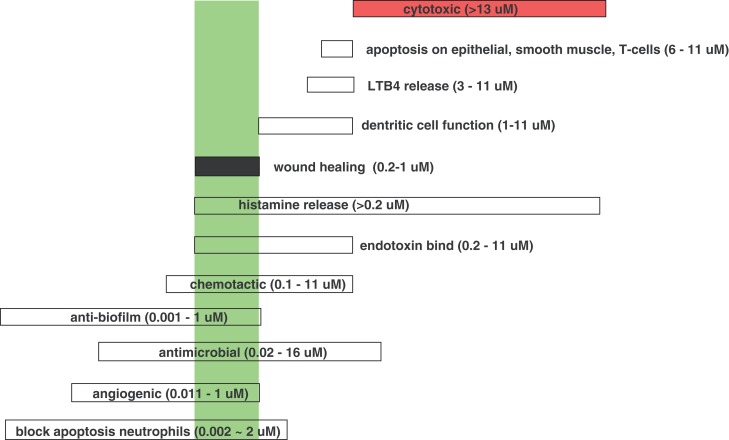
**Effective concentrations for the various, different activities of LL-37**. (Data from Kai-Larsen and Agerberth, 2008). The green box indicates the wound-healing concentration range of LL-37.

The interactions of LL-37 with host cells are numerous and complex and not completely understood. LL-37 was shown to have a chemotactic effect on inflammatory cells, and depending on its concentration has shown a remarkable ability to modulate their effects. As previously mentioned, hCAP-18/LL-37 is stored predominantly in human neutrophil granules, but also in T cells, monocytes, lymphocytes, natural killer cells, B cells, and mast cells. In wounds, LL-37 is secreted in wound fluid and sweat, and is upregulated in response to infection. It is interesting that at different concentrations LL-37 can have opposing effects on host cells. For example, LL-37 exposures that were at or below ∼1 μM enhanced neutrophil survival and increased fibroblast migration and proliferation. In contrast, higher concentrations were cytotoxic (enhanced apoptosis) and amplified an acute inflammatory response (Oudhoff et al., 2010). Reported activities and effects of LL-37 at particular concentration levels are shown in Figure 7. As development of these peptides continues it is important to view the overall effects of LL-37 at 0.2 ∼1 μM, concentrations at which wound-healing effects are observed.

In addition, shorter peptides may also prove useful to retain antimicrobial activity and potentially have less host-toxicity. The cathelicidin LL-37 and shorter derivatives were tested for toxicity (oto-toxicity, primary skin irritation/corrosion, acute eye irritation, and toxicity by repeated dose administration in rats), and no toxicity was found for P60.4, a 24aa acetylated and amidated peptide derivative of LL-37 (Nell et al., 2006).

Mechanistically, LL-37 has been shown to use formyl protein receptor like 1 (FPRL-1), a cell surface GPCR, to chemoattract human peripheral blood neutrophils, monocytes, and T cells (Yang et al., 2000). The suppression of neutrophil apoptosis below 1 μM was shown to occur through the activation of FPRL-1 and P2 × 7, and antagonists of these receptors attenuated the suppression (Nagaoka et al., 2006). By direct activation of the P2 × 7 receptor, LL-37 stimulated IL-1β secretion from monocytes (Elssner et al., 2004). In line with the P2 × 7 receptor mechanism, LL-37 induced pore formation and release of intracellular ATP as evidenced by the uptake of the fluorescent nucleic acid dye, YO-PRO-1. Known inhibitors of the P2 × 7 receptor (KN04, KN-62, and oxidized ATP) inhibited IL-1β processing and release induced by LL-37 in a dose-response fashion. It was noted that this effect did not appear to be due to increased endogenous ATP since (a) this increase in response to LL-37 was only in the 100–200 nM ATP range (mM ATP levels are needed to activate IL-1β release) and (b) apyrase, which catalyzes the hydrolysis of ATP, completely inhibited exogenously added ATP (5 mM), but did not block IL-1β release from LL-37 stimulated cells (Wewers and Sarkar, 2009).

On a molecular level, LL-37’s ability to insert deeply into host cell membranes may support the hypothesis that the functional interaction between LL-37 and P2 × 7 involves transmembrane segment-mediated binding. This may also explain the finding that d-LL-37 has similar activity to LL-37 in modulating the P2 × 7 receptor (Tomasinsig et al., 2008) and at inhibiting *Pseudomonas* biofilm formation (Dean et al., 2011a), especially since the hydrophobic environment of the membrane allows for specific interactions to be formed between polypeptides irrespective of their chirality.

In the presence of Gram-negative bacteria, LL-37 was able to interact directly with LPS to reduce its binding to LBP, MD2, or other components of the TLR4 receptor complex, thus reducing activation of the downstream pathway (Scott et al., 2000). LL-37 also inhibited LPS-induced translocation of the NF-kB subunits p50 and p65, and selectively modulated gene transcription, completely inhibiting certain pro-inflammatory genes (p50, TNFAIP2) and reducing the expression of others (TNF-α) (Bowdish et al., 2004). LL-37 was also shown to directly trigger MAPK pathways that can in turn impact pro-inflammatory pathways. In other reports, LL-37 has been shown to activate mast cells via mas-related Gene X2 (MrgX2, a novel GPCR) (Subramanian et al., 2011), and may act as a functional ligand for CXCR2 on human neutrophils (Zhang et al., 2009). Thus, LL-37 has a diverse and potentially advantageous immune-modulating effect on host cells within the wound environment.

### LL-37 and wound healing

The resistance of chronic wounds to heal has been shown to be associated with the presence of multispecies pathogenic biofilms. As previously stated, once biofilm is broken down, the underlying bacterial colonies can be targeted by the immune system as well as by antibiotics, potentially allowing recovery of the otherwise non-healing wound. LL-37 is capable of performing all of these functions (anti-biofilm, antimicrobial, immune-modulating), and when administered topically, can potentially avoid the many hurdles of systemic peptide delivery.

An intriguing aspect of LL-37 with respect to skin wounds is its interaction with keratinocytes. Keratinocytes, the predominant cell type found in the epidermis, form barriers against microbial pathogens during wound closure, and keratinocyte migration is an important step in skin wound healing. Growth factors important to wound healing (IGF-1 and TGF-α) induced the expression of hCAP-18/LL-37 in human keratinocytes (Sørensen et al., 2003) and the P2 × 7–SFK–Akt–CREB/ATF1 signaling pathway activated by LL-37 in keratinocytes was established (Nijnik et al., 2012). In a Boyden chamber assay, LL-37 (1 μg/ml) induced the maximum level of keratinocyte migration, and this was shown to progress via EGFR transactivation (Tokumaru et al., 2005). LL-37 was also found to protect human keratinocytes from apoptosis via the activation of the COX-2 pathway (Chamorro et al., 2009). hCAP-18 is strongly expressed in healing skin epithelium, and treatment with antibodies raised and affinity purified against LL-37 inhibited re-epithelialization (wound closure) in a concentration-dependent manner (Heilborn et al., 2003). Adenovirus-mediated LL-37 gene transfer was found to promote wound healing in diabetic ob/ob mice by increasing the re-epithelialization rate and granulation tissue formation (Carretero et al., 2008). *In vivo*, cathelicidin-deficient mice were shown to be more susceptible to group A *Streptococcus* infection compared to normal mice, supporting the involvement of epithelial cell-derived cathelicidin in host immune defense (Braff et al., 2005).

Fibroblasts, another epidermal cell, also play a key role in tissue repair because they change their phenotype during the late phase of repair and begin to proliferate and synthesize large amounts of extracellular matrix which is crucial for wound resolution. LL-37 can induce fibroblast proliferation, and the stimulation of fibroblast growth by LL-37 was inhibited by KN-62 (P2 × 7R antagonist), supporting a role for the P2 × 7R pathway (Tomasinsig et al., 2008) in this process.

In other models, LL-37 stimulated the healing of mechanically induced wounds in monolayers of human epithelial lung cells (NCI-H292 cells) (5 μg/ml) and in differentiated primary airway epithelium (1 μg/ml). These effects were shown to be mediated through epidermal growth factor receptor, a GPCR, and MAP/extracellular signal-regulated kinase (ERK) (Shaykhiev et al., 2005). Similar signaling was reported within oncology research, where LL-37 was found to have affinity to the insulin-like growth factor 1 receptor (IGF-1R) resulting in phosphorylation of IGF-1R with downstream signaling of the mitogen-activated protein kinase/ERK pathway (Girnita et al., 2012). In summary, LL-37 induces signal transduction in the host cells that may contribute to the cellular processes involved in promoting wound healing.

## Clinical Development

The use of topical cationic peptides to treat bacterial infection has precedent and in recent years there have been a number of AMPs entering clinical trials. For example, the peptide omiganan (MBI 226), a 12-residue amide derivative of indolicidin (a cathelicidin isolated from bovine neutrophils), is in late-stage development as a topical antimicrobial for the prevention of local catheter-site infections (Ross et al., 2007). Peptide mimetics of AMPs (Flemming et al., 2009) have also progressed into clinical trials. Lytixar (LTX 109) is a synthetic antimicrobial peptidomimetic currently in phase II trials for the topical treatment of infections of multi-resistant bacterial strains (Isaksson et al., 2011). Another example, PMX-30063, is a defensin mimetic that is currently being evaluated in patients with acute bacterial skin infections (Morrisey et al., 2012). PMX-30063 showed potent activity against the Gram-positive bacteria tested, particularly *Staphylococci*, and the activity was unaffected by resistance to existing antibiotics, including MDR *Staphylococci*. Lactoferrin, another AMP, has shown efficacy against *Pseudomonas* biofilms (Kamiya et al., 2012). Treatment of *Pseudomonas* biofilm with lactoferrin in combination with xylitol led to both structural disruption of the preformed biofilm, as well as a reduction of viable bacteria through membrane permeabilization (Ammons et al., 2009, 2011). The RIP has shown effectiveness against severe polymicrobial infections (Lopez-Leban et al., 2010). Although no data has yet been published, LL-37 itself is currently in early development for the treatment of hard-to-heal wounds and a clinical study in venous leg ulcer patients is scheduled (Development Program LL-37, 2012). Thus, the use of peptides as a therapeutic approach for chronic infected wound treatment is beginning to be explored in the clinic, and this novel approach will hopefully lead to new and effective therapies for these difficult to treat conditions. Incorporation of anti-biofilm peptides or their synthetic derivatives in therapeutic topical applications may improve outcomes for infections ranging from chronic wounds, burns, implanted medical devices, and pneumonia.

## Conclusion and Development Challenges

Novel treatments for chronic wounds, pneumonia, and medical implant-associated infections are critically needed. These infections are often characterized by polymicrobial infections mediated by biofilm-forming bacteria, including *P. aeruginosa* (James et al., 2008). Desired characteristics of a novel therapeutic for these wounds would include a broad-spectrum, anti-biofilm treatment that is capable of withstanding the host environment, including protease and wound-exudate secretions.

Overall, LL-37 and other AMPs (Chalekson et al., 2002; Hirsch et al., 2009) appear to be promising for the treatment of chronically infected wounds since their anti-biofilm properties coupled with the combination of host and pathogen effects should act in harmony to expose and clear the underlying bacteria, and the peptide interaction with keratinocytes and fibroblasts should encourage wound closure. Proteolytic cleavage and systemic toxicity are two concerns with the development of peptides, but in the case of wound treatment, topical administration should lower these hurdles, especially in light of LL-37’s demonstrated stability in wound exudate. We previously demonstrated the effectiveness of d-LL-37 to inhibit biofilm formation of *S. aureus* and *P. aeruginosa*, two common pathogens found in chronic infected wounds (Dean et al., 2011a,b). d-LL-37 represents a potential therapeutic candidate by being a protease-resistant peptide that is effective in inhibiting biofilm formation, increasing the rate of twitching motility, and possesses potentially wound-healing properties toward the host, illustrating its potential to be developed as topical treatments against biofilm-forming bacteria in skin wounds. The concentration-dependent effects of LL-37 ranges from anti-biofilm and an ability to block neutrophil apoptosis at low nM levels, to antimicrobial and chemotactic effects at 0.1 ∼ 10 μM levels, to cytotoxic effects at levels above 10 μM. Thus, a challenge to the development of AMPs for the treatment of chronic wounds may lie in defining the optimal efficacious concentrations of the peptide within the wound environment. The design of new peptides with a larger therapeutic index between wound-healing properties and eukaryote cytotoxicity is warranted, and indeed this is one direction that the field is going. Other hurdles to market that remain for AMP therapeutics include cost-of-goods and the design of AMPs with pharmacokinetic properties that maintain optimal drug exposure levels at the target site of infection. To that end, since AMP therapeutics may be effective as an add-on to current therapy, they should also be evaluated for their effectiveness in the presence of standard antibiotics (Brandenburg et al., 2012). It is clear that the data to date for LL-37 as a potential treatment for infected wounds is encouraging. The use of multiple concurrent strategies to treat these wounds is likely to be most effective, combining physical debridement, systemic antibiotics, and topical treatments such as peptides that are able to reduce biofilm.

## Conflict of Interest Statement

The authors declare that the research was conducted in the absence of any commercial or financial relationships that could be construed as a potential conflict of interest.
